# Diagnostic potential of circulating LncRNAs in human cardiovascular disease: a meta-analysis

**DOI:** 10.1042/BSR20181610

**Published:** 2018-12-21

**Authors:** Fei Luo, Tao Wang, Lini Zeng, Shanshan Zhu, Wenjun Cao, Wei Wu, Hongfu Wu, Tangbin Zou

**Affiliations:** 1 Dongguan Key Laboratory of Environmental Medicine, School of Public Health, Guangdong Medical University, Dongguan 523808, China; 2 The Third Affiliated Hospital of Guangdong Medical University (Longjiang Hospital of Shunde District), Foshan 528318, China; 3 School of Basic Medical Sciences, Guangdong Medical University, Dongguan 523808, China; 4 School of Basic Medical Sciences, Southern Medical University, Guangzhou 510515, China

**Keywords:** biomarker, Cardiovascular disease, Diagnosis, Long non-coding RNA

## Abstract

Cardiovascular disease (CVD) is a major killer of the human population around the world. Identifying effective diagnostic biomarkers for CVDs is particularly important in order to guide optimizing treatment. Accumulating evidence on aberrantly regulated circulating long non-coding RNAs (LncRNAs) promise to serve as a diagnostic or prognostic biomarker for various types of CVDs. We summarized studies to identify the potential diagnostic values of LncRNAs in CVD patients. We included articles reporting on the association between LncRNAs and diagnosis in CVDs. We calculated sensitivities, specificities, and area under the curves of LncRNAs. The pooled overall sensitivity and specificity for LncRNAs expression profile in differentiating CVD patients from controls (non-CVDs or healthy subjects) were 0.74 (95%CI 0.68–0.80) and 0.81 (95%CI 0.76–0.85), respectively; the overall positive likelihood ratio, 3.9 (95%CI 3.1–4.9); the negative likelihood ratio, 0.32 (95%CI 0.25–0.40); corresponding to an area under curve of 0.85 (95%CI 0.82–0.88) and overall diagnostic odds ratio 12 (95%CI 9–18). Subgroup analysis showed that the detection of LncRNAs expression in plasma substantially improved the diagnostic accuracy. Likewise, meta-regression analysis indicated that the detection method and sample size were the main source of heterogeneity. All these results suggested a relatively good reference value of LncRNAs as auxiliary biomarkers for CVDs, and should be considered in cases where the diagnosis is uncertain. Population-based prospective cohort studies are warranted to confirm our findings.

## Introduction

Cardiovascular disease (CVD) is the leading cause of death and disease burden around the world. It is estimated that approximately 100 million American adults (>1 in 3) have ≥1 type of CVD. A total of 11.5% of American adults (27.6 million) have been diagnosed with heart disease. By the year 2030, 43.9% of the U.S. population is predicted to have some form of CVD [1]. With CVDs being such a huge burden, advancements in disease management have been directed toward not only the treatment of such diseases, but also the development of platforms for early detection and the possible prevention of CVDs.

The term circulating long non-coding RNA (LncRNA) defines transcripts lacking coding features longer than 200 nucleotides and has been proposed in recent years as a modulator of cancer pathways and biomarkers of cancer outcomes [2–4]. Meanwhile, these molecules are increasingly acknowledged as non-invasive and readily accessible biomarker for diagnostic and prognostic applications of various CVDs [5–7]. Many researchers recently assessed the diagnostic and prognostic value of LncRNAs in elderly patients with CVDs, including stroke, myocardial infarction (MI), coronary artery disease (CAD) and heart failure (HF) [8–11]. The pervasiveness of the bloodstream and its perfusion through all organs and tissues enables various biomolecules generated and released locally, to be distributed throughout the circulation. Altered concentrations of these molecules, like LncRNAs in blood, have been linked to various disease states, including that of CVDs [12]. For instance, a LncRNA, named LIPCAR (long intergenic ncRNA predicting cardiac remodeling) [10] that was down-regulated in an early phase after the cardiac event and remained elevated during later stages; UCA1 (urothelial carcinoma-associated 1) was down-regulated shortly after AMI, but was found to be increased when assessed 3 days after cardiac injury [13]. MYHEART (myosin heavy chain associated RNA transcript) [14,15] was found to be elevated in AMI patients compared with control subjects and positively correlates with the cardiac injury marker cardiac troponin T. The above examples are only a fraction of the several instances in which LncRNAs have demonstrated promising diagnostic potential. Because deaths from CVDs are among the leading causes of disease deaths around the world, there is an emphasis to identify earlier and less invasive stages of CVDs to achieve better clinical outcomes.

Taken together, the aim of this meta-analysis is to comprehensively explore the accuracy of LncRNA-based biomarkers in the diagnosis of cardiovascular outcomes, to demonstrate its role as non-invasive diagnostic biomarker in this setting.

## Methods

### Search strategy

We identified relevant retrospective or prospective published studies following PRISMA guidelines (Preferred Reporting Items Systematic Reviews and Meta-Analysis) for appropriate articles, by using the following terns: (a) ‘Long non-coding RNA’ or LncRNA and (b) ‘cardiovascular disease’ or ‘coronary artery disease’ or ‘myocardial infarction’ or ‘heart failure’ or stroke and (c) diagnosis or diagnostic or sensitivity or specificity or ‘receiver operating characteristic curve’. The bibliographic references of retrieved articles were equally systematically reviewed for additional studies of LncRNAs in patients with CVDs. Meanwhile, the records were screened assessing titles and abstracts and thereafter retrieved in full text and judged according to eligibility criteria.

### Study selection

All eligible studies in this meta-analysis were required to satisfy the following criteria: (a) studies patients were diagnosed with cardiovascular events (including stroke, myocardial infarction, coronary heart disease and heart failure); (b) expression of LncRNA was measured in blood (serum, plasma, peripheral whole blood and leukocyte in peripheral blood); (c) the study investigated the association between LncRNAs expression and clinical outcomes; (d) cohort studies and case–control studies were included and (e) published in English. The exclusion criteria were: (a) duplicate studies; (b) without complete data to tabulate 2×2 table; (c) had an unclear definition of the control group(s) and (d) non-English, non-human studies, reviews, meta-analyses, letters etc.

### Data extraction and quality assessment

Data were extracted separately by both of us from included studies into a specially designed spreadsheet (Excel, version 14.7.0; Microsoft Corp), information was collected concerning the basic aspects of the study. For each study, the numbers of true-positive, false-positive, true-negative and false-negative LncRNAs findings in the diagnosis of CVDs were recorded. The quality of the studies was assessed according to the Quality Assessment of Diagnostic Accuracy Studies (QUADAS-2) checklist. All studies were independently analyzed by two reviewers and any disagreement in respect of study eligibility, data extraction or methodological quality assessment was resolved through consensus.

### Statistical analysis

All statistical analysis were performed using Stata (12.0 Stata Corp, College Station, TX) software and Review Manager (RevMan, Version 5). The sensitivity, specificity, positive and negative likelihood ratio (LR+ and LR-) and diagnostic odds ratio (DOR) of each LncRNAs associated with the diagnostic value of CVD events were calculated from individual studies. Area under the curve (AUC) calculated on the summary receiver operating characteristic (sROC) curves were used as a measure of the overall performance of the diagnostic accuracy of LncRNAs in distinguishing CVD patients from controls. Heterogeneity between studies was assessed with the Cochran *Q*-test and the Inconsistency Index (*I*^2^). A random effect model (Der Simonian and Laird) was used for the meta-analysis if there was heterogeneity caused by non-threshold effect (*P*<0.1 or *I*^2^>50%), otherwise a fixed effect model (Mantel–Haenszel) would be applied (*P*>0.1 or *I*^2^<50%). Subgroup analysis, sensitivity analysis and meta-regression were conducted to explore the potential sources of heterogeneity caused by non-threshold effect. In addition, we utilized Deeks’ funnel plot asymmetry test to assess publication bias of selected studies. *P*<0.05 indicated significance.

## Results

### Literatures search and studies’ characteristics

**Figure 1:**
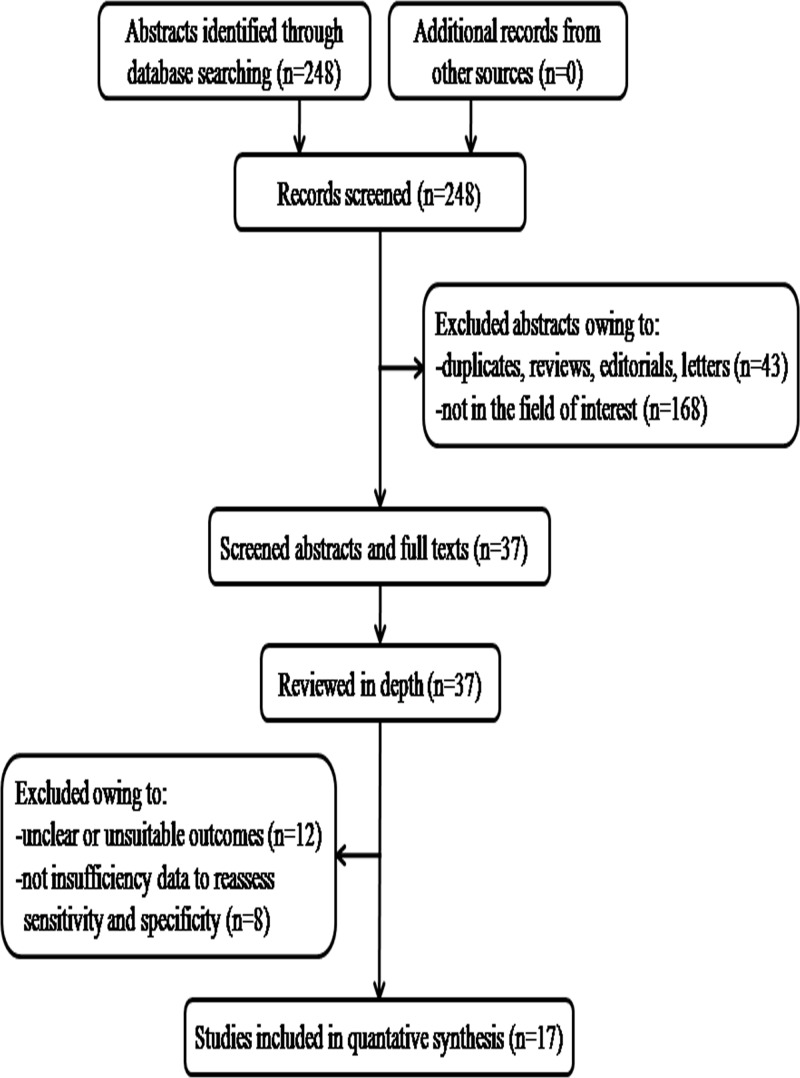
A flow diagram demonstrating the study selection process

The comprehensive computer literature search of PubMed, MEDLINE and the Cochrane database identified 248 references (Figure 1). Removal of duplicates, commentary, letters and basic research articles and yielded 37 full-text articles after reviewing of titles and abstracts. Subsequently, 20 full-text articles were excluded from the analysis due to insufficient data to reassess sensitivity and specificity. No additional studies were found on screening the references of these articles. Finally, 17 articles including data on the diagnostic performance of LncRNAs in cardiovascular events were eligible for inclusion [13,16–31] (Table 1).

**Table 1:** Characteristics of the 17 articles included in the meta-analysis

Study	year	CVD	Patients (controls)	Source of control	Specimen	Method	Design type	LncRNA	Reference standard
Yin [16]	2017	CAD	30(30)	Healthy	Plasma	qPCR	Retrospective	GAS5 (D)	CAG
Li [17]	2017	CAD	137(115)	Healthy	Blood	qPCR	Prospective	Upperhand (U)	CAG, SYNTAX scores, with >50% organic stenosis, ECT
Zhang [18]	2017	CAD	300(180)	Non-CAD	Plasma	qRT-PCR	Retrospective	LIPCAR, H19 (U)	CAG, with >50% organic stenosis, echocardiography, Gensini score
Zhang [19]	2016	CAD	99(30)	Healthy	Serum	qPCR	Retrospective	uc022bqs.1 (U)	CAG and clinical manifestations
Cai [20]	2015	CAD	211(171)	Non-CAD	PBMC	qPCR	Retrospective	LncPPAR (U)	CAG
Yang [21]	2015	CAD	221(187)	Non-CAD	Plasma	qPCR	Retrospective	Coromarker (U)	CAG, with ≥50% organic stenosis
Xu [22]	2017	CAD	102(89)	Non-CAD	Blood	qRT-PCR	Retrospective	IFNG-AS1 (U)	CAG, with ≥50% organic stenosis
Cai [23]	2016	CAD	211(171)	Non-CAD	PBMC	qPCR	Retrospective	Coromarker (U)	CAG, with≥50% organic stenosis
Yan [13]	2016	MI	49(15)	Non-AMI	Plasma	qPCR	Retrospective	UCA1 (U)	cTnI, CK-MB, pathological Q wave and ST-segment elevation or depression
Zhang [24]	2016	MI	103 (149, 95)	Non-AMI (149)	Blood	qRT-PCR	Retrospective	ZFAS1 (D)	Ischemic symptom plus increased cTnI and CK-MB, pathological Q wave and ST-segment elevation or depression
				Healthy (95)				CDR1AS (U)	
Meng [25]	2018	MI	47(43)	Healthy	Blood	qPCR	Retrospective	APPAT (D)	Ischemic symptom plus increased cTnI and CK-MB, pathological Q wave
Li [26]	2018	MI	46(40)	Healthy	Blood	qRT-PCR	Retrospective	LIPCAR (U)	Ischemic symptoms, significantly elevated myocardial enzymes (cTnI and CK-MB), elevated ST-segment of ECG, pathological Q wave and narrowing ≥50% in the left main coronary artery and ≥70% in one or several of the major coronary arteries. echocardiography, PCI
Xuan [27]	2016	HF	72(60)	Non-HF	Plasma	qRT-PCR	Retrospective	MHRT, NRON (U)	2000 JESC/ACCC guidelines the redefinition of myocardial infarction and 2007 NACB guidelines for the diagnosis and treatment of acute coronary syndromes
Yu [28]	2017	HF	67(67)	Non-HF	Plasma	qPCR	Retrospective	UCA1 (U)	Typical clinical symptoms, LVE ≤40%, BNP ≥35 pg/ml
Wang [29]	2017	IS	36(25)	Healthy	Plasma	qPCR	Retrospective	H19 (U)	Routine biochemical tests, CMRI
Feng [30]	2018	IS	126(125)	Non-IS	Plasma	qRT-PCR	Retrospective	ANRIL (D)	Routine biochemical tests, WHO criteria, CMRI
Zhu [31]	2017	IS	189(189)	Healthy	PBL	qPCR	Retrospective	MIAT (U)	Experienced their first IS with symptom onset within 24 h, WHO criteria

Abbreviations: BNP, brain natriuretic peptide; CAD, coronary artery disease; CAG, coronary angiography; CK-MB, creatine kinase-MB; CMRI, cerebral magnetic resonance imaging; cTnI, cardiac troponin I; CVD, cardiovascular disease; ECT, emission computed tomography; HF, heart failure; IS, ischemic stroke; LncRNA, long non-coding RNA; LVEF, left ventricular ejection fraction; MI, myocardial infarction; PB, peripheral blood; PBL, peripheral blood leukocytes; PBMC, peripheral blood mononuclear cell; PCI, percutaneous coronary intervention; qPCR, real-time polymerase chain reaction; qRT-PCR, RT-PCR/qPCR combined technique; RT-PCR: reverse transcription-polymerase chain reaction; D, down-regulated; U, up-regulated.

All of these included studies were performed from 2015 to 2018, involving 3827 participants and all were from China (Supplementary Table S1). Among them, 8 papers studied CADs, 4 studied MIs, 3 studied strokes and 2 studied HFs. The main detection method for 16 different LncRNAs expression was quantitative real-time PCR (qPCR). About 12 LncRNAs were up-regulated and 4 (GAS5, ZFAS1, APPAT and ANRIL) were down-regulated. The specimen sources were mostly plasma from non-CVD patients or healthy subjects. The QUADAS-2 assessment indicated that the majority (>80%) of studies were at high risk of bias, and all had concerns regarding applicability. The primary reason is the blinding of participants and personnel (performance bias) (Supplementary Figure S1).

### Diagnostic performance

**Figure 2:**
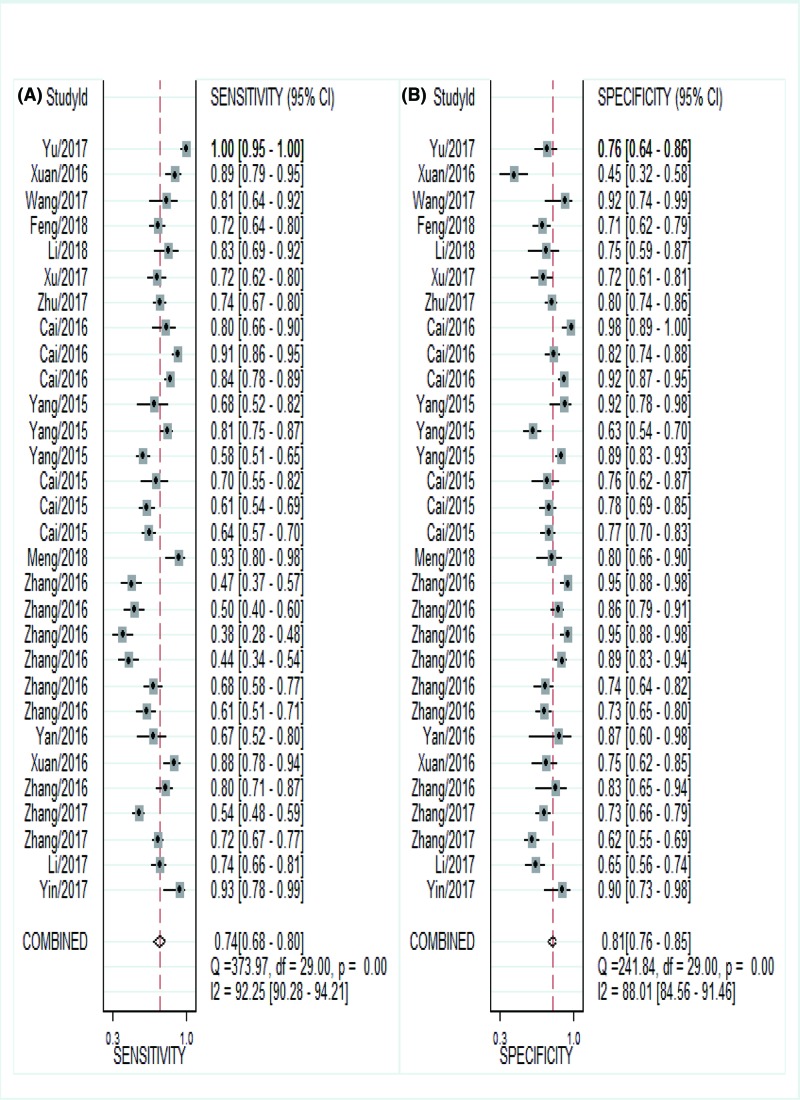
Forest plots for studies on overall LncRNAs used in the diagnosis of CVDs among 30 studies included in the meta-analysis (**A**) sensitivity and (**B**) specificity.

**Figure 3:**
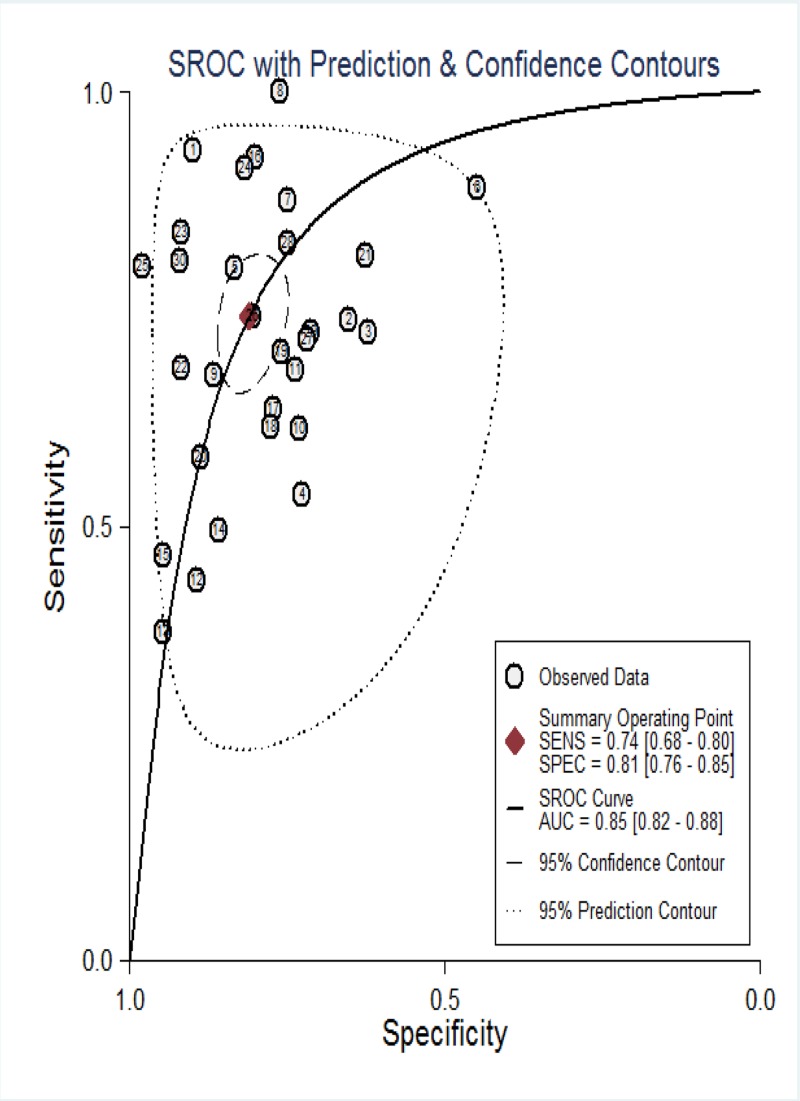
Summary receiver operator characteristic curves (SROC) of LncRNAs for the diagnosis of CVDs in overall population

Currently, our objective was to summarize the results of individual studies and understand the potential utility of LncRNAs in the diagnosis of CVDs. About 30 studies from 17 articles utilizing LncRNAs reported adequate cardiovascular events and were included in the meta-analysis. The Spearman correlation coefficient was 0.191 (*P*=0.312), suggesting no notable threshold effect in the accuracy estimate of LncRNAs. Forest plots of the sensitivity and specificity of LncRNAs for diagnosing CVDs are displayed in Figure 2. A significant heterogeneity was observed (*I*^2^ = 92.25% and *I*^2^ = 88.01%). And thus, the random-effects model was used to calculate the pooled effect. The indexes are as follows: sensitivity, 0.74 (95%CI 0.68–0.80); specificity, 0.81 (95%CI 0.76–0.85); the pooled LR+, LR- and DOR were 3.9 (95%CI 3.1–4.9), 0.32 (95%CI 0.25–0.40), 12 (95%CI 9–18), respectively. In addition, we plotted the sROC curve to evaluate diagnostic accuracy (Figure 3), AUC was 0.85 (95%CI 0.82–0.88), indicating a good diagnostic accuracy of overall LncRNAs in detecting CVDs. To explore the potential sources of heterogeneity between studies, we conducted a series of analyses, including subgroup analysis, sensitivity analysis, meta-regression and publication bias.

### Subgroup analysis

We undertook many subgroup analyses, including disease type, detection method, sample size and more (Supplementary Table S2). Moreover, the sROC curve was plotted to evaluate diagnostic accuracy (Supplementary Figure S2). In terms of disease type, the lower diagnostic value was found in patients with MI (DOR 9 (95%CI 6–13)) than CAD (DOR 13 (95%CI 7–22)). Compared with specimen of blood, LncRNAs have a higher overall diagnostic accuracy in plasma, with sensitivity of 0.63 (95%CI 0.52–0.72) versus 0.80 (95%CI 0.69–0.88), specificity of 0.82 (95%CI 0.74–0.88) versus 0.77 (95%CI 0.68–0.84), LR of 3.4 (95%CI 2.6–4.5) versus 3.5 (95%CI 2.5–5.0), NLR of 0.46 (95%CI 0.37–0.57) versus 0.26 (95%CI 0.16–0.41), DOR of 8 (95%CI 5–10) versus 14 (95%CI 7–27) and AUC of 0.79 (95%CI 0.76–0.83) versus 0.85 (95%CI 0.82–0.88), respectively. Hierarchical analysis based on detection method suggested that qPCR trials significantly reported a higher rate of identifying CVD patients compared with qRT-PCR. The pooled sensitivity 0.80 (95%CI 0.72–0.86) versus 0.65 (95%CI 0.55–00.74), specificity 0.83 (95%CI 0.78–0.87) versus 0.78 (95%CI 0.69–00.84), LR+ 4.7 (95%CI 3.5–6.3) versus 2.9 (95%CI 2.3–3.7), LR- 0.24 (95%CI 0.18–0.34) versus 0.45 (95%CI 0.37–0.55), DOR 19 (95%CI 12–33) versus 6 (95%CI 5–9) and AUC 0.89 (95%CI 0.85–0.91) versus 0.78 (95%CI 0.74–0.81), hinting that qPCR may be a better matrix for the analysis of LncRNAs in conforming CVDs. In detected samples from healthy, results were 0.74 (95%CI 0.63–0.83) for sensitivity, 0.84 (95%CI 0.76–0.90) for specificity, 4.6 (95%CI 3.2–6.8) for PLR, 0.31 (95%CI 0.21–0.45) for NLR, 15 (95%CI 9–26) for DOR and 0.87 (95%CI 0.84–0.90) for AUC. In the non-CVD samples, sensitivity, specificity, PLR, NLR, DOR and AUC were 0.74 (95%CI 0.66–0.82), 0.84 (95%CI 0.74–0.84), 3.6 (95%CI 2.8–4.6), 0.33 (95%CI 0.23–0.44), 11 (95%CI 7–17) and 0.84 (95%CI 0.80–0.87), suggesting that LncRNA from controls with healthy rather than non-CVDs has a higher diagnostic accuracy. There were noticeable differences in DOR among different subgroups of detection method and sample size, and others also have a slight difference.

### Sensitivity analysis, meta-regression analysis and publication bias

**Figure 4:**
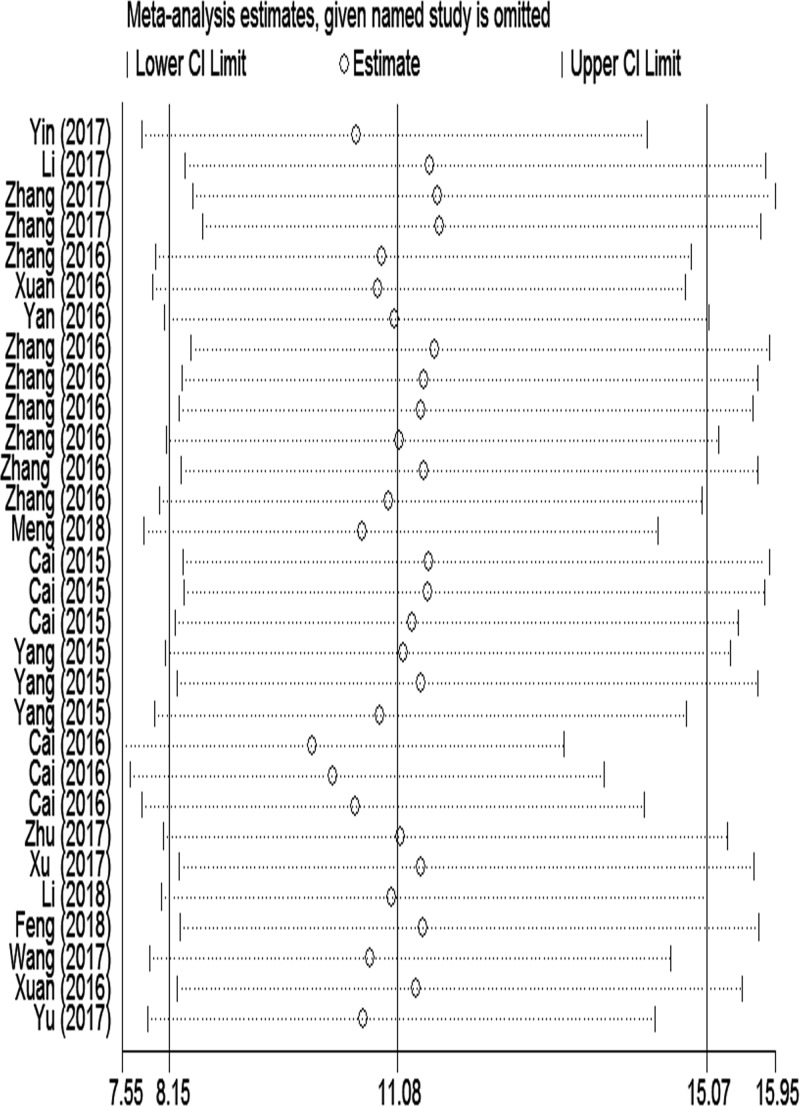
Sensitivity analysis of the result of the meta-analysis for CVDs

**Figure 5:**
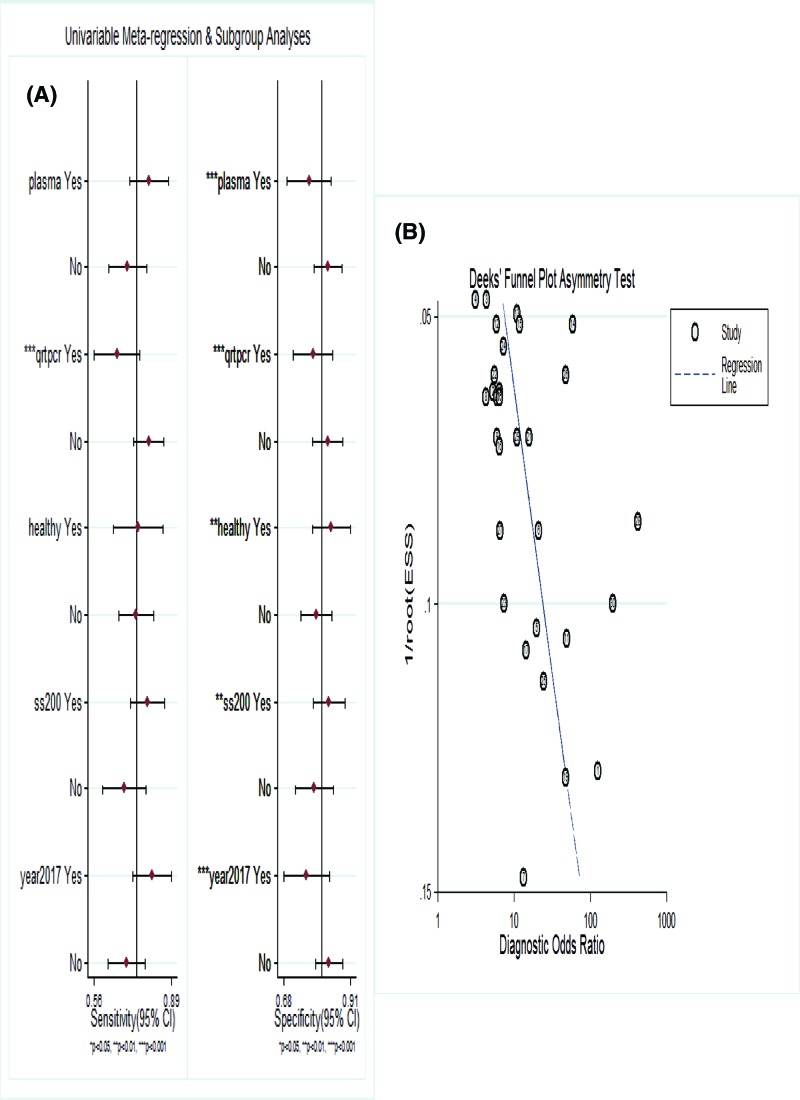
Assessment of the heterogeneity of LncRNAs for inclusion studies (**A**) Univariable meta-regression for sensitivity and specificity of LncRNAs for diagnosis of CVDs. (**B**) Deeks’ funnel plot evaluating the potential publication bias of the included studies.

Sensitivity analysis was performed to assess the contribution of each study to the pooled estimate by excluding individual studies one at a time and recalculating the pooled OR estimates for the remaining studies. We found that excluding any one study did not substantially change the pooled OR in the result of meta-analysis of CVDs (Figure 4). We read those studies again and conducted meta-regression analysis on the bias of source of control, specimen, detection method, sample size and publication year (Figure 5A). We found that specificity was influenced by source of control, specimen, detection method, sample size and publication year, whereas sensitivity was affected only by detection method. The LncRNA detected by qPCR shows a higher sensitivity and specificity in the diagnosis of CVDs. Formal tests for publication bias have also carried out using Deeks’ test. The *P* value was 0.02 suggesting a potential publication bias (Figure 5B).

## Discussion

CVDs account for high morbidity and mortality worldwide. Despite extraordinary efforts in cardiovascular research, the early clinical diagnosis and treatments for CVDs have not been dramatically improved. As a novel class of endogenous transcripts, non-coding RNAs (ncRNAs) are recently emerging as important regulators of cellular processes with many implications in human diseases [32]. Among multiple ncRNAs, LncRNA has emerged as a novel biomarker in various diseases, whereas the diagnostic ability of LncRNAs in CVDs is unclear. Recent studies suggest critical roles of LncRNAs in modulating the initiation and progression of cardiovascular diseases. The association between the dynamic alterations of LncRNAs and clinical outcomes has to be explored furthermore. To our knowledge, this is the first meta-analysis on the diagnostic accuracy of LncRNAs for CVDs.

In this meta-analysis, we thoroughly searched multiple databases and retrieved 17 articles, including 30 studies pertaining to the diagnostic value of LncRNAs for cardiovascular events. Our data showed aberrantly expressed LncRNAs in CVDs, with 12 up-regulated and 4 down-regulated. The pooled effect sizes for diagnosis revealed that LncRNA signature harbored moderate sensitivity of 0.74 (95%CI 0.68–0.80) and high specificity of 0.81 (95%CI 0.76–0.85). The AUC of 0.85 (95%CI 0.82–0.88) for differentiating patients with CVDs from controls (non-CVD patients or healthy subjects), which means that circulating LncRNAs represent a promising diagnostic marker for CVDs [33]. Pooled PLR was 3.9, indicating that the probability of CVDs increased by 3.9‐fold with positive LncRNAs testing. Moreover, NLR was 0.32, implying that the probability of CVDs increased by 68% when the studied LncRNAs were negative [34]. Importantly, the pooled DOR was estimated to be 12, which is larger than 1.0, also showed a powerful capacity of LncRNAs for CVDs diagnosis [35]. All these results indicated that LncRNAs achieved a relatively good diagnostic efficacy in the management of CVDs, and therefore could be developed as additional or independent biomarker to aid in CVDs diagnosis. However, the quality of included studies was assessed by QUADAS and the result varied from moderate to high, which may influence the stability of pooled results. Furthermore, previous studies that were included in the current meta-analysis were mostly retrospective studies and small sample size of CVDs. Therefore, further prospective studies with large sample size and combination of other candidate molecular markers should be required to confirm these findings.

In addition, due to the large degree of heterogeneity observed among included studies, and we also try to well interpret the causes from different aspects. Significant heterogeneity in sensitivity and specificity was still present in all subgroup analyses (all *P*<0.05, Supplementary Table S2). According to our results, the heterogeneity was decreased in some subgroups. The less heterogeneity was detected in studies with a smaller sample size and in studies detected by qPCR. Second, subgroup analyses were conducted based on CVDs found that the lower diagnostic value of LncRNAs in patients with MI (DOR 9 (95%CI: 6–13)) than CAD (DOR 13 (95%CI: 7–22)). Third, the source of controls could be mainly divided into healthy subjects and non-CVD controls. We found the diagnostic accuracy of LncRNAs in both subgroups was relatively high, suggesting LncRNAs could serve not only as screening biomarkers but also as biomarkers to distinguish patients from non-CVD controls. Fourth, plasma (DOR 14 (95%CI: 7–27)) were mostly used in detecting LncRNAs as specimen, and which had better diagnostic accuracy than blood (DOR 8 (95%CI: 5–10)) in the diagnosis of cardiovascular outcomes. A subsequent meta-regression was carried out, indicated LncRNA profiling would influence specificity rather than sensitivity. The results suggested that the specimen (*P*=0.18), source of control (*P*=0.47), publication year (*P*=0.09) were not the origin of the heterogeneity. By contrast, the detection method and sample size significantly affected the diagnostic accuracy for CVDs. For publication bias, a statistically significant value (*P*=0.02) in the Deeks’ funnel plot indicated potential publication bias, and the limited number of studies for analysis on CVDs could be a possible source of the publication bias. Nevertheless, our sensitivity analysis identified no outlier studies, hinting that our results were relatively reliable.

Although our efforts to accomplish a comprehensive and accurate analysis, there were still some limitations in meta-analysis. First, substantial heterogeneity was detected among the included studies, which may be a potential source of bias in the meta-analysis. This heterogeneity may be caused through methodological diversity among the different studies. Second, the baseline differences among the patients in the included studies and the study quality may also contribute to the heterogeneity of the results. Since all the enrolled participants were Asians from China in the study, which reduced the applicability of the results across different races. Third, publication bias was discovered by Deeks’ test for the result of the included studies. Our meta-analysis included a limited number of studies of which 16 studies (16/30) had a study population of 200 participants or less. A study investigating the effect of small trials in 13 meta-analyses found that small studies tend to have a more beneficial treatment effect [36]. The limited number of studies for analysis on CVDs could be a viable source of the publication bias. Fourth, the way to calculate or extract data from the receiver operating characteristic curves might be less reliable, compared with those directly obtained from original articles.

Taken together, the early diagnostic accuracy of a test is a measure of clinical effectiveness, and increased accuracy does result in improved patient outcomes. Our research demonstrated that LncRNAs could serve not only as screening biomarkers but also as biomarkers to distinguish patients from non-CVD controls in early diagnosis. Additional studies are needed to validate the feasibility of LncRNAs as next-generation biomarkers, reliable and reproducible, in cardiovascular outcomes.

## Supporting information

**Figure S1:** Overall quality assessment of eligible studies by QUADAS-2 tool. A. Methodological quality summary. B. Methodological quality graph.

**Figure S2:** Summary receiver operator characteristic (SROC) curves based onLncRNAs in Subgroup analyses. A. CAD; B. MI; C. Blood; D. Plasma; E.Healthy; F.Non-CVDs.

**Table S1:** Characteristics of eligible studies included in the meta-analysis.

**Table S2:** Assessment of diagnostic accuracy and heterogeneity in subgroup analysis.

## Abbreviations

AUC: area under the curve
CAD: coronary artery disease
CVD: cardiovascular disease
DOR: diagnostic odds ratio
HF: heart failure
LncRNA: long non-coding RNA
MI: myocardial infarction
ncRNA: non-coding RNA
qPCR: quantitative reverse transcription PCR
sROC: summary receiver operating characteristic
