# Supplementary material for: Diagnostic potential of circulating LncRNAs in human cardiovascular disease: a meta-analysis
Source: Biosci Rep. 2018 Dec 21;38(6):BSR20181610. doi: 10.1042/BSR20181610 (PMC6435511; doi:10.1042/BSR20181610)
Supplement: Supplementary file 1 [file bsr20181610_Supp1.pdf]

Table S1. Characteristics of eligible studies included in the meta-analysis.

Author	Year	LncRNAs	TP	FP	FN	TN	SEN%	SPE%	AUC
Yin	2017	GAS5	28	3	2	27	93.1	88.8	0.9783
Li	2017	Upperhand	101	40	36	75	73.7	65.2	0.728
Zhang[1]	2017	LIPCAR	217	68	83	112	72.2	62.3	0.722
Zhang[2]	2017	H19	161	49	139	131	53.6	73	0.631
Zhang	2016	uc022bqs.1	79	5	20	25	79.5	83.3	0.895
Xuan[1]	2016	MHRT	64	33	8	27	88.3	44.4	0.702
Xuan[2]	2016	NRON	63	15	9	45	84.1	75.5	0.865
Yu	2017	UCA1	67	16	0	51	100	76.12	0.89
Yan	2016	UCA1	33	2	16	13	67.3	85.2	0.757
Zhang[1]	2016	ZFAS1	63	40	40	109	61.1	73	0.664
Zhang[2]	2016	ZFAS1	70	25	33	70	68.2	73.7	0.732
Zhang[3]	2016	CDR1AS	45	16	58	133	43.6	89.3	0.671
Zhang[4]	2016	CDR1AS	39	5	64	90	37.7	94.78	0.657
Zhang[5]	2016	ZFAS1+CDR1AS	51	21	52	128	49.6	86	0.691
Zhang[6]	2016	ZFAS1+CDR2AS	48	5	55	90	46.4	94.7	0.752
Meng	2018	APPAT	37	10	3	40	78.72	93.02	0.9302
Cai[1]	2015	LncPPAR	134	39	77	132	63.7	76.9	0.727
Cai[2]	2015	LncPPAR	99	27	62	94	61.8	77.4	0.712
Cai[3]	2015	LncPPAR	35	12	15	38	70	75.9	0.769
Yang[1]	2015	coromarker	128	21	93	166	58	89	0.795

Yang[2]	2015	coromarker	146	56	34	94	81.1	62.9	0.796
Yang[3]	2015	coromarker	28	3	13	34	68.29	91.89	0.811
Cai[1]	2016	coromarker	177	14	34	157	84	92	0.92
Cai[2]	2016	coromarker	147	22	14	99	91	81.9	0.905
Cai[3]	2016	coromarker	40	1	10	49	80	98	0.96
Zhu	2017	MIAT	140	37	49	152	74.1	80.4	0.842
Xu	2017	IFNG-AS1	73	25	29	64	71.6	72.1	0.755
Li	2018	LIPCAR	38	10	8	30	82	75	0.782
Feng	2018	ANRIL	91	36	35	89	72.2	71.2	0.759
Wang	2017	H19	29	2	7	23	80.6	92	0.91

Abbreviations: TP, true positive; FP, false positive; FN, false negative; TN, true negative;SEN, sensitivity；SPE, specificity; AUC, area under the curve.

Table S2. Assessment of diagnostic accuracy and heterogeneity in subgroup analysis.

Subgroups	N	SEN(95%CI)	SPE(95%CI)	LR+(95%CI)	LR-(95%CI)	DOR(95%CI)	AUC(95%CI)
**All**	30	0.74(0.68-0.80),I^2=92.25, P<0.001	0.81(0.76-0.85),I^2=88.01, P<0.001	3.9(3.1-4.9)	0.32(0.25-0.40)	12(9-18)	0.85(0.82-0.88)
**CVD**							
CAD	15	0.75(0.68-0.80),I^2=91.97, P<0.001	0.81(0.74-0.86),I^2=89.28, P<0.001	3.9(2.8-5.5)	0.31(0.24-0.41)	13(7-22)	0.84(0.81-0.87)
MI	9	0.61(0.49-0.73),I^2=88.81, P<0.001	0.85(0.77-0.90),I^2=83.14, P<0.001	4.0(2.9-5.5)	0.46(0.35-0.60)	9(6-13)	0.82(0.79-0.85)
others	6	0.87(0.74-0.94),I^2=85.85, P<0.001	0.74(0.62-0.83),I^2=86.56, P<0.001	3.4(2.2-5.0)	0.18(0.08-0.37)	19(7-49)	0.86(0.83-0.89)
**Method**							
qPCR	17	0.80(0.72-0.86),I^2=89.99, P<0.001	0.83(0.78-0.87),I^2=84.80, P<0.001	4.7(3.5-6.3)	0.24(0.18-0.34)	19(12-33)	0.89(0.85-0.91)
qRT-PCR	13	0.65(0.55-0.74),I^2=91.67, P<0.001	0.78(0.69-0.84),I^2=89.74, P<0.001	2.9(2.3-3.7)	0.45(0.37-0.55)	6(5-9)	0.78(0.74-0.81)
**Speciman**							
blood	10	0.63(0.52-0.72),I^2=90.19, P<0.001	0.82(0.74-0.88),I^2=86.44, P<0.001	3.4(2.6-4.5)	0.46(0.37-0.57)	8(5-10)	0.79(0.76-0.83)
plasma	12	0.80(0.69-0.88),I^2=93.38, P<0.001	0.77(0.68-0.84),I^2=89.44, P<0.001	3.5(2.5-5.0)	0.26(0.16-0.41)	14(7-27)	0.85(0.82-0.88)
others	8	0.77(0.69-0.83),I^2=89.93, P<0.001	0.84(0.78-0.88),I^2=76.40, P<0.001	4.8(3.3-6.9)	0.27(0.19-0.39)	17(9-34)	0.88(0.85-0.90)
**Sample size**							
<200	16	0.79(0.69-0.86),I^2=90.62, P<0.001	0.84(0.77-0.89),I^2=86.90, P<0.001	4.9(3.4-7.1)	0.25(0.17-0.36)	20(12-34)	0.89(0.86-0.91)
≥200	14	0.69(0.61-0.76),I^2=92.80, P<0.001	0.79(0.73-0.83),I^2=88.88, P<0.001	3.2(2.5-4.1)	0.40(0.31-0.50)	8(5-12)	0.81(0.77-0.84)
**Source of control**							
healthy	10	0.74(0.63-0.83),I^2=92.16, P<0.001	0.84(0.76-0.90),I^2=85.22, P<0.001	4.6(3.2-6.8)	0.31(0.21-0.45)	15(9-26)	0.87(0.84-0.90)
non-CVD	20	0.74(0.66-0.82),I^2=92.59, P<0.001	0.79(0.74-0.84),I^2=89.03, P<0.001	3.6(2.8-4.6)	0.33(0.23-0.44)	11(7-17)	0.84(0.80-0.87)
**Year**							
~2017	19	0.70(0.62-0.77),I^2=92.64, P<0.001	0.84(0.78-0.88),I^2=89.95, P<0.001	4.3(3.2-5.7)	0.36(0.29-0.45)	12(8-18)	0.84(0.81-0.87)

2017~	11	0.82(0.71-0.89),I^2=92.83, P<0.001	0.75(0.70-0.80),I^2=79.21, P<0.001	3.3(2.5-4.3)	0.24(0.15-0.41)	13(6-28)	0.82(0.78-0.85)

Abbreviations: CVDs, cardiovascular diseases; CAD, coronary artery disease; MI, myocardial infarction;SEN, sensitivity；SPE, specificity; CI, confidence interval; LR+, positive likelihood ratio; LR-, negative likelihood ratio; DOR, diagnostic odds ratio; AUC, area under the curve.

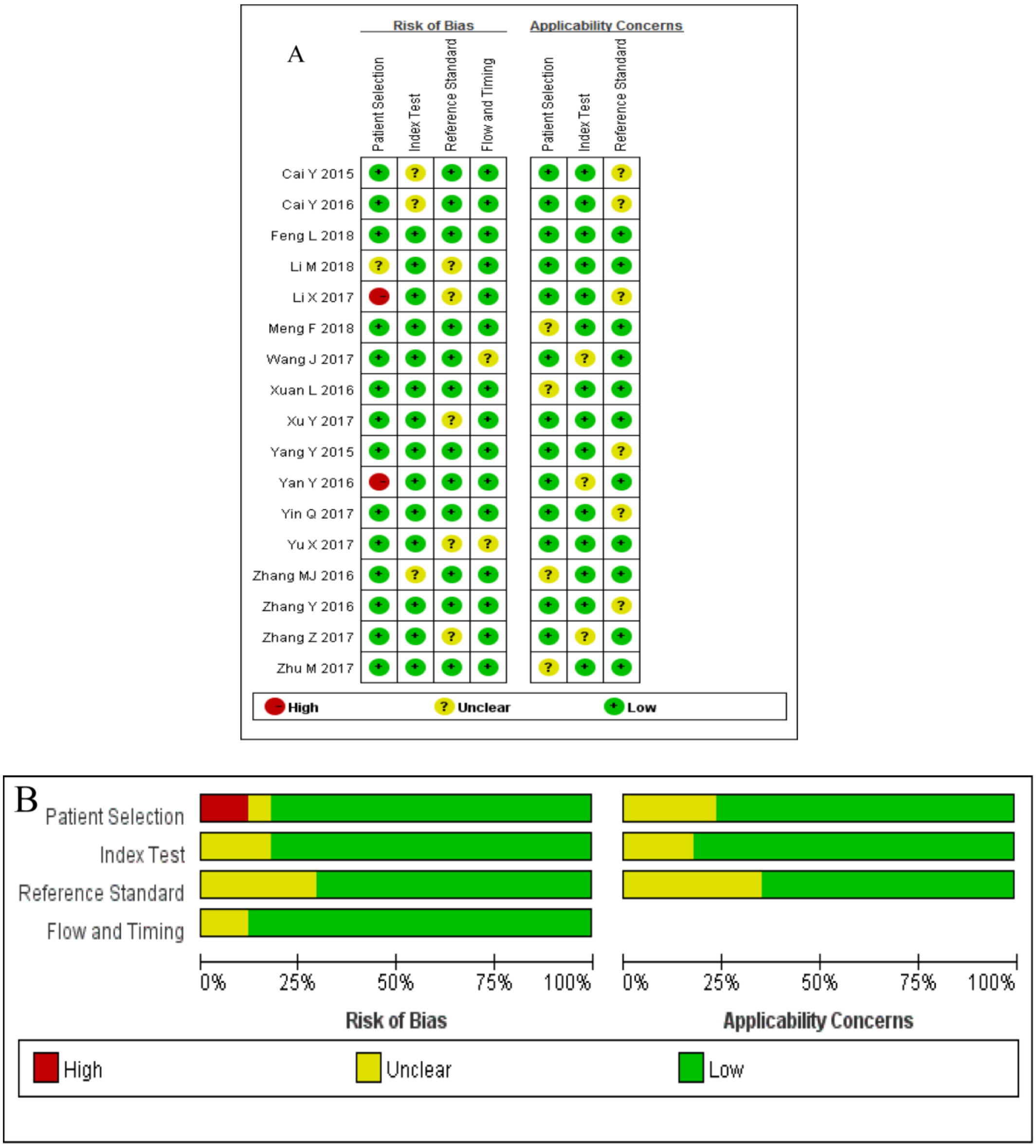

Figure S1. Overall quality assessment of eligible studies by QUADAS-2 tool. A. Methodological quality summary. B. Methodological quality graph.

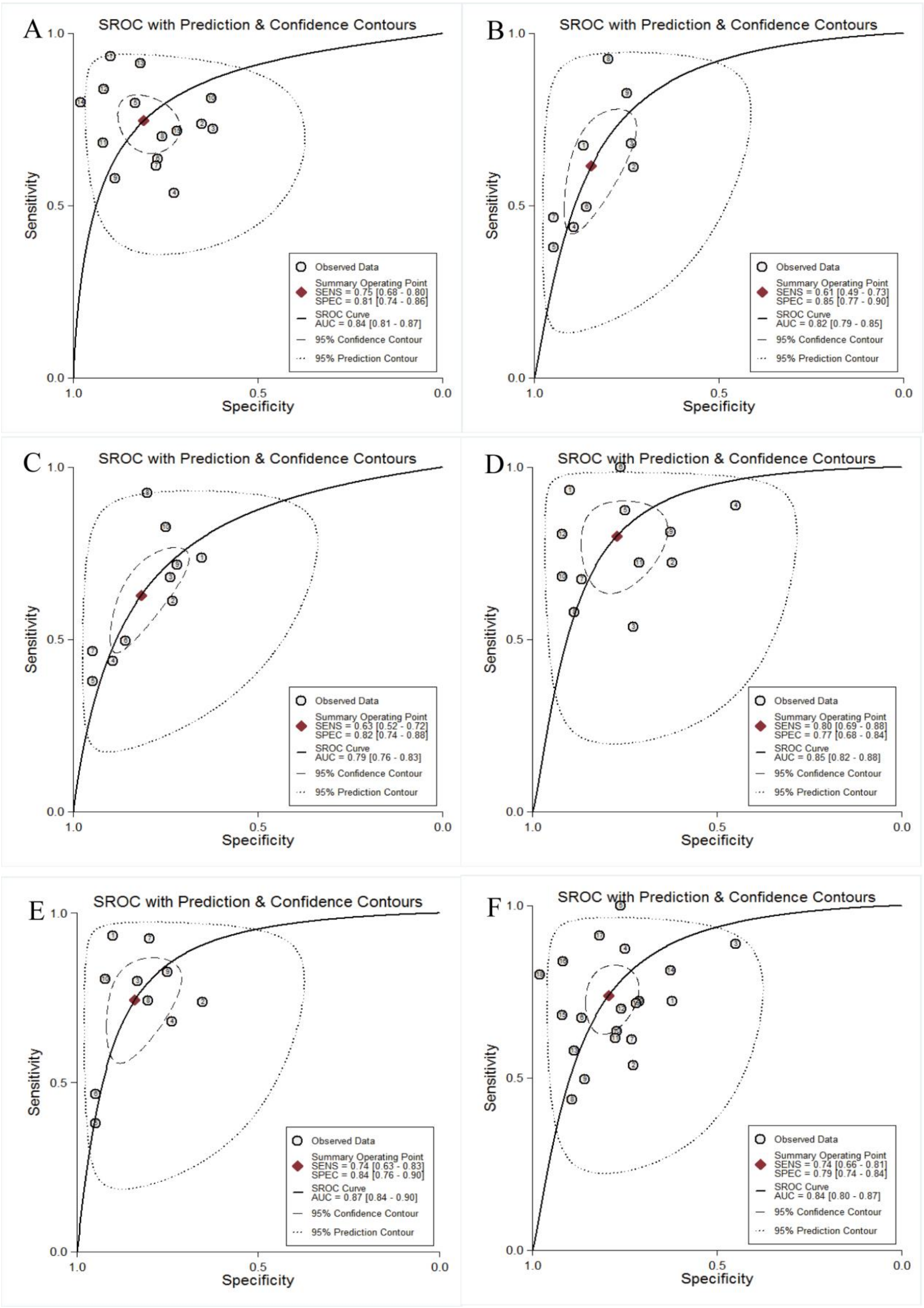

Figure S2. Summary receiver operator characteristic (SROC) curves based onLncRNAs in Subgroup analyses. A. CAD; B. MI; C. Blood; D. Plasma; E.Healthy; F.Non-CVDs.
